# The Aryl Hydrocarbon Receptor in Energy Balance: The Road from Dioxin-Induced Wasting Syndrome to Combating Obesity with Ahr Ligands

**DOI:** 10.3390/ijms22010049

**Published:** 2020-12-23

**Authors:** Nathaniel G. Girer, Craig R. Tomlinson, Cornelis J. Elferink

**Affiliations:** 1 Department of Pharmacology and Toxicology, The University of Texas Medical Branch at Galveston, Galveston, TX 77550, USA; ngirer89@gmail.com; 2 Department of Molecular and Systems Biology, Norris Cotton Cancer Center, Dartmouth Hitchcock Medical Center, Dartmouth College, Lebanon, NH 03756, USA; craig.r.tomlinson@dartmouth.edu

**Keywords:** aryl hydrocarbon receptor, energy metabolism, obesity

## Abstract

The aryl hydrocarbon receptor (AHR) has been studied for over 40 years, yet our understanding of this ligand-activated transcription factor remains incomplete. Each year, novel findings continually force us to rethink the role of the AHR in mammalian biology. The AHR has historically been studied within the context of potent activation via AHR agonist 2,3,7,8-tetrachlorodibenzo-*p*-dioxin (TCDD), with a focus on how the AHR mediates TCDD toxicity. Research has subsequently revealed that the AHR is actively involved in distinct physiological processes ranging from the development of the liver and reproductive organs, to immune system function and wound healing. More recently, the AHR was implicated in the regulation of energy metabolism and is currently being investigated as a potential therapeutic target for obesity. In this review, we re-trace the steps through which the early toxicological studies of TCDD led to the conceptual framework for the AHR as a potential therapeutic target in metabolic disease. We additionally discuss the key discoveries that have been made concerning the role of the AHR in energy metabolism, as well as the current and future directions of the field.

## 1. Introduction

Almost 50 years have passed since Dr. Alan Poland’s initial reports on the discovery of the aryl hydrocarbon receptor (AHR) [1], and nearly 7000 manuscripts concerning the AHR have been published on the subject of this ligand activated transcription factor (as of writing). The AHR is an important basic helix-loop-helix member of the Per-ARNT-SIM family of proteins that binds to a wide array of compounds. In the absence of ligand binding, the AHR is contained in the cytosol within a protein complex composed of X-associated protein 2, heat shock protein 90, and p23 [2]. Binding of agonists (e.g., 2,3,7,8-tetrachlorodibenzo-*p*-dioxin, TCDD) to the AHR generally results in nuclear translocation of the cytosolic complex and subsequent dissociation of AHR from the complex to form a heterodimer with aryl hydrocarbon receptor nuclear translocator (ARNT). The AHR-ARNT heterodimer then binds to canonical 5′-GCGTG-3′ sequences known as xenobiotic response elements (XRE) to activate gene transcription. While the majority of AHR-driven effects are known to occur via this mechanistic pathway, additional physiological mechanisms of action have been described to date. For example, the AHR can bind to alternative, “non-canonical” DNA sequences via the formation of a DNA-binding heterodimer with Kruppel-like factor 6 (KLF6), and can also act directly as an E3 ubiquitin ligase [3,4].

Despite decades of research on the AHR, our understanding of the AHR remains incomplete. Every year presents more novel mechanisms of AHR action, newly characterized ligands, and additional endogenous roles for the receptor. One relatively new area of research on AHR biology that we shall focus upon in this review is the ever expanding role of the AHR in regulating energy balance/metabolism. What follows is a historical perspective of how the AHR became recognized as an important endogenous regulator of energy balance and energy metabolism in the body, and how research arrived at this conclusion despite the historically negative reputation of the AHR due to its involvement in dioxin toxicity. In line with the theme of this Special Issue on AHR biology, we hope to portray a discrete pathway via which early toxicological studies of TCDD provided the rationale for exploiting the AHR as a therapeutic target in obesity. Along the way, we shall highlight key discoveries in our understanding of how the AHR influences energy balance, and will summarize the current and future directions of this particular area of AHR biology.

## 2. Part I: TCDD, AHR, and the Wasting Syndrome

The story of how the AHR became an important aspect of energy metabolism originates, as much of AHR research does, in early studies on the relationship between TCDD and human toxicity. In 1971, physician Dr. Alan Poland began investigating why workers from 2,4,5-trichlorophenol (2,4,5-T)-producing factories were developing chloracne and porphyria cutanea tarda [5]. That seminary work sent Dr. Poland down a road that, with the help of Dr. Andrew Kende, led to the eventual identification of an “inducible receptor” we now know as the aryl hydrocarbon receptor [1]. By the time of Dr. Poland’s work, the potential health hazards of TCDD, a contaminant of 2,4,5-T herbicide production, were known, and TCDD had been established as the main driver of chloracne in exposed individuals [6,7]. Subsequent work in Dr. Poland’s laboratory further established a role for TCDD in mediating porphyria cutanea tarda [8,9]. However, Dr. Poland’s work in 1971 also importantly noted that “…among the many symptoms exhibited by the factory workers were anorexia and weight loss” [5]. Subsequent animal studies from multiple laboratories soon confirmed that TCDD induces weight loss and reduced food intake, a condition coined “wasting syndrome” [10,11,12,13]. These studies thus provided the first links between TCDD, energy balance, and the yet to be discovered AHR.

As the initial characterization of the AH locus and the AHR protein developed throughout the 1970s and 1980s, the mechanism via which TCDD induces wasting syndrome in animals was also under close investigation. By 1980, two studies had provided evidence that in monkeys and rats, TCDD alters serum concentrations of triglycerides, free fatty acids, and cholesterol [14,15], and by the middle of the decade, another pair of papers from Dr. Seefeld’s group had proposed that TCDD caused wasting syndrome through establishing a decreased “set point” for body weight that is achieved through reduced food and energy intake [16,17]. A few years later, Lakshman et al. (1988) discovered that TCDD reduces liver and adipose fatty acid synthesis rates, and more specifically, a reduction in the activities of the fatty acid synthase (Fas), acetyl-coA carboxylase (Acaca), and 3-hydroxy-3-methylglutaryl-coenzyme A reductase enzymes [18,19]. Weber et al. (1991) likewise determined that TCDD also reduced the activity of key gluconeogenic enzymes in the liver, including phosphoenolpyruvate carboxykinase and glucose-6-phosphatase [20]. By the late 1990s, TCDD was further revealed to suppress fat cell differentiation and adipogenesis, providing yet another mechanism for TCDD-induced wasting syndrome [21,22,23]. By the turn of the century, a mechanism had emerged for how TCDD exposure led to the wasting syndrome, presumably through the actions of the AHR. Surprisingly perhaps, modern research still continues to uncover additional ways by which TCDD induces wasting syndrome via the AHR, one example being through the regulation of TCDD-inducible poly(adenosine diphosphate-ribose) polymerase gene expression [24,25].

Ethical constraints on exposing humans to TCDD ensured that all of the studies on TCDD toxicity throughout the 1970s and 1980s were performed in lab animals. The relevance of these animal data to humans thus remained conjectural. Epidemiological studies throughout the 1990s would eventually provide a concrete connection between TCDD and human toxicity. However, these studies focused primarily on examining cancerous endpoints [26,27,28,29], and apart from the role of TCDD in mediating the wasting syndrome in lab animals, whether or not TCDD influences energy metabolism in humans remained somewhat a mystery. In 1997, Henriksen et al. published data collected from a cohort of Vietnam War veterans involved in Operation Ranch Hand indicating that dioxin exposure was adversely associated with type II diabetes, glucose metabolism, and insulin production [30]. Subsequent studies further confirmed an increased risk for diabetes and impaired insulin signaling with increasing TCDD exposure in other cohorts with known exposures [31,32,33,34,35,36]. These studies are important because they confirmed that the AHR can influence metabolism in humans, and as is discussed below, eventually provided a rationale for exploring AHR antagonists as a therapeutic treatment for obesity.

By the start of the 21st century, scientists had developed a good understanding of the adverse effects of TCDD exposure in humans and the molecular mechanism of AHR action. However, the specific gene pathways that linked TCDD exposures to human toxicities remained largely unknown. The transcriptomics technologies we have today were only just being realized toward the end of the 20th century; thus, merely a handful of genes were known to be regulated by the AHR at the time [37,38,39,40,41]. Then in 2000, two independent laboratories began the first attempts to link specific gene pathways to TCDD-driven human toxicities by employing then-novel microarray technologies in a human hepatoma cell line treated with TCDD [42,43]. While both studies found TCDD clearly influenced several gene pathways (e.g., cell proliferation, cell signaling, drug metabolism), neither study could link specific transcriptomic changes elicited by TCDD exposure to the adverse health consequences of TCDD exposure. These studies nevertheless were important footholds to begin testing hypotheses for the underlying genetic mechanisms connecting TCDD to human disease. Regarding the role of the AHR in energy metabolism, several studies emerged in the mid-2000s that revealed novel connections between TCDD and the modulation of specific metabolic gene pathways such as glucose metabolism, cholesterol biosynthesis, lipogenesis, and bile acid metabolism [44,45,46,47]. Those studies confirmed that the previously observed TCDD-elicited changes in metabolic enzyme activities are indeed linked to changes in gene transcripts. However, a role for the AHR in mediating these TCDD-driven changes again remained elusive, as none of these studies analyzed the effects of TCDD in the absence of AHR. These missing data were soon to be acquired as the first *Ahr*-null mouse models came to fruition in the mid-1990s. In fact, the generation of these mice would lead to a full-blown transformation into how the AHR was studied, as discussed in the following section.

## 3. Part II: AHR Transgenic Mouse Studies and the Age of Endogenous AHR Biology

Concurrent with the microarray revolution, another revolution had begun within the field of AHR biology in the mid-1990s. By then, researchers had recognized that certain dietary compounds could variously activate or inhibit AHR-driven monooxygenase activity [48,49,50], but the idea that such compounds could activate AHR in a therapeutic manner (we now know they can [51,52,53]) had not yet taken hold. This concept first arose from manuscripts such as one from Dr. Christopher Bradfield’s laboratory in 1991 that examined indole-3-carbinol (I3C), a compound found in Brassica vegetables. Their team deemed the compound as non-toxic, despite being a high affinity agonist for the AHR [48]. At around the same time, scientists also began to categorize certain AHR ligands as “endogenous”, and recognized that AHR ligands could be produced via normal physiological processes. While the AHR field currently lacks a consensus definition for “endogenous AHR ligands”, they are generally viewed as compounds whose origins are not from external sources or associated with industrial chemical syntheses (e.g., TCDD). Endogenous ligands may also be considered as distinct from naturally derived compounds such as I3C, which originates exogenously as a part of the normal human diet. An early usage of the term “endogenous” can be found in a publication from Perdew and Pabbs (1991), who determined that gut bacteria present in rat fecal suspensions were able to transform tryptophan into chemical derivatives that activated the AHR [54]. Heath-Pagliuso et al. would later characterize two of these compounds, tryptamine and indole acetic acid, in 1998 [55]. In fact, these initial insights into endogenous AHR ligands in the gut have recently blossomed into an entire branch of study of AHR biology focused around host-gut interactions. We now know that tryptophan is the source and/or precursor for many endogenous AHR ligands, and that many of these gut-derived AHR ligands have been shown to have distinct roles in regulating intestinal health and the immune response [56]. Recent work has also revealed how the gut microbiota also influence host metabolic processes, such as the intestinal production of short-chain fatty acids [57].

Subsequent to the first reports of endogenous AHR ligands, further questioning of the association between AHR activation and adverse health outcomes would later continue with the development of an *Ahr*-null mouse. Between 1995 and 1997, three laboratories independently generated and characterized mice lacking *Ahr* expression [58,59,60]. Gonzalez and his team accomplished this by replacing the first exon of *Ahr* with a neomycin gene, and found that the loss of *Ahr* expression impaired immune system function and disrupted proper development of the liver. Another group led by Fujii-Kuriyama in Japan alternatively replaced the first exon with the bacterial gene beta-galactosidase fused to a nuclear localization signal, and observed malformation of the cleft palate and kidneys in mouse embryos. The third *Ahr*-null mouse, generated by Bradfield and colleagues, was accomplished via complete deletion of exon 2, and revealed that AHR loss produced a plethora of hepatic defects, further cementing a role for the AHR in the development of the liver. Importantly, all three of these studies focused on how the loss of AHR signaling created numerous developmental defects, and thus provided the foundations to explore the physiological role of the AHR, independent of its activation by environmental toxicants.

Utilizing these novel AHR knockout mouse models, studies soon revealed that the AHR was surprisingly involved in a wide array of physiological processes including development of the hepatic vasculature [61], cardiovascular physiology [62], wound healing [63], and the development of the reproductive organs [64]. A physiological role for the AHR in energy metabolism would not surface until nearly a decade after the generation of the first AHR knockout mice however. The first evidence that the AHR endogenously regulates energy metabolism appeared in studies that examined the effects of constitutive AHR activation. In 2010, Lee et al. generated a mouse model expressing a constitutively-active form of AHR (CA-AHR) via deletion of the minimum ligand-binding domain [65]. Their data demonstrated that mice expressing this form of AHR spontaneously develop hepatic steatosis, and attributed this outcome to increased fatty acid import (particularly via CD36), suppression of fatty acid oxidation, inhibited fatty acid export, increased oxidative stress, and increased mobilization of peripheral fat stores. The following year, research from the laboratory of Shelley Tischkau demonstrated that global *Ahr* loss enhanced insulin sensitivity and glucose tolerance, and reduced activation of the peroxisome proliferator-activated receptor alpha (PPARα) pathway [66]. The PPARα pathway regulates fatty acid oxidation and glucose metabolism; consistent with the observation that fatty acid oxidation was increased by constitutively active AHR, loss of *Ahr* led to a decrease in fatty acid oxidation. Tischkau and colleagues later expanded upon these findings, and observed that in mice fed a high-fat diet, systemic *Ahr* loss increased energy expenditure and resulted in decreased adiposity [67]. They further found that loss of the AHR was able to preserve insulin sensitivity in high-fat diet-challenged mice. Most recently, researchers employing AHR knockout mouse models have also uncovered a role for the AHR in Crohn’s disease and adenine-driven kidney disease [68,69]. All of these studies brought forth evidence that the AHR is actively involved in energy metabolism, and the idea that the AHR could be utilized to target these gene pathways in a therapeutic manner was soon to be realized.

## 4. Part III: Antagonist Theory: AHR Inhibition as a Means for Treating Obesity

Concomitant with the rapidly evolving concept of endogenous AHR activity in the 1990s was the recognition that certain AHR ligands could inhibit TCDD activation of the AHR. As research characterizing the AHR protein and its involvement in TCDD toxicity matured, Dr. Stephen Safe and his colleagues at Texas A&M University discovered a whole new class of AHR ligands that bound to the AHR with high affinity, but were poor activators of AHR activity as measured through the induction of benzo[a]pyrene hydroxylase and ethoxyresorufin O-deethylase [70]. They further observed that some of these compounds had the ability to block TCDD induction of these enzymes, and thus their group defined a new class of AHR ligands known as “antagonists”. Dr. Safe’s group would later go on to discover several more of these compounds in subsequent studies [71,72,73], as would other laboratories [74,75,76]. Given the intense focus of the AHR as mediating TCDD toxicity at the time, the discovery of AHR antagonists that block TCDD-driven enzymatic activity naturally led to the hypothesis that AHR antagonists could be utilized to prevent TCDD toxicity. Indeed, such hypotheses were soon tested in mouse models of TCDD exposure, and met with success [77]. AHR antagonists have since exhibited such success that a few have found themselves in the drug pipelines of pharmaceutical companies, including Bayer, Magenta Therapeutics, Celgene, and JAGUAHR Therapeutics.

The notion of targeting AHR biology to treat metabolic disease did not arise from the use of antagonists however, and instead developed in response to keen observations made between mice that express an AHR variant with low affinity for ligands (*Ah^d^*) and mice that express an AHR variant with high affinity for ligands (*Ah^b^*). These two *Ahr* gene variants, originally discovered during the 1970s [78], differ by approximately 10-fold in TCDD binding affinity. Accordingly, expression levels of target genes, such as *Cyp1a1* and *Cyp1b1*, are expressed 10-fold lower in mice harboring the *Ah^d^* variant. Employing mice that express either variant to identify a potential role for the AHR in obesity, the Tomlinson laboratory in 2012 revealed that the differences in AHR activation in these mice significantly affected body weight, relative fat mass, liver physiology, and liver gene expression when the mice were fed a fat/sugar/cholesterol/salt/protein-rich Western diet [79]. Following up on those findings, their group also tested two mechanistically different AHR antagonists, alpha-naphthoflavone (αNF) [80,81] and CH-223191 [77,82], to further delineate the role of the AHR in obesity. They discovered that the inhibition of AHR signaling by systemic administration of either antagonist significantly reduced obesity and adiposity, and that liver steatosis was attenuated to near-control levels. Furthermore, they showed that regardless of AHR ligand affinity, inhibition of the AHR was highly effective in preventing obesity and liver steatosis in both male and female mice [83,84].

Concomitant with the discovery that AHR inhibition was protective against obesity, the question as to how the AHR is activated in diet-induced obesity was still under investigation. Utilizing in vivo mouse studies and in vitro mouse hepatocyte experiments, Moyer et al. (2016) showed that diet-derived low-density lipoproteins induced toll-like receptor 2/4 to trigger downstream signaling events including indoleamine-2,3-dioxygenase 1 (IDO1) activation via NF-κB [83]. IDO1 is an AHR target gene that metabolizes tryptophan to kynurenine (Kyn), a well-known AHR agonist [85,86,87,88], and therefore, likely participates in an IDO1-AHR-IDO1 positive feedback loop. Consequently, Moyer et al. (2016) proposed that a sustained increase in Kyn-induced AHR activity derived from the excess consumption of Western diet helped to promote the obese phenotype. This hypothesis was further tested rigorously in vivo by the Tomlinson group, who subsequently showed that the addition of Kyn to a low-fat diet induced AHR activity in mice to cause weight gain, fatty liver, and hyperglycemia (Rojas et al. 2020, in press) [89]. Consistent with their previous findings, cytochrome P450 1B1 (CYP1B1) and stearoyl-coA desaturase 1 (SCD1) appeared to act as downstream effectors of Kyn-induced AHR signaling.

Having shown that activation of the AHR promoted obesity, and AHR inhibition prevented obesity, a remaining key question was whether AHR inhibition could also reverse obesity and its associated comorbidities. This question was answered recently in a study by Rojas et al. (2020). Their data specifically demonstrated that obese C57BL6/J mice maintained on a Western diet, when switched to a Western diet containing the AHR antagonist αNF, lost body mass to a degree in which the mass of these mice nearly matched that of control mice maintained on a low-fat diet [90]. Inhibition of the AHR in the diet-switched mice also reversed fatty liver disease, decreased PPARα activity, and reduced CYP1B1, SCD1, and secreted phosphoprotein 1 (SPP1) expression, all of which are positively correlated with the obese state. The culmination of work performed by Tomlinson and colleagues during this past decade ultimately provides strong evidence that inhibition of the AHR could be a highly effective therapeutic strategy for the treatment of obesity and associated illnesses, particularly through its action on PPARα, SCD1, and/or SPP1. Indeed, that idea continues to remain an important area of research within their laboratory.

## 5. Part IV: AHR Regulation of Energy Metabolism: Current and Future Directions

**Figure 1:**
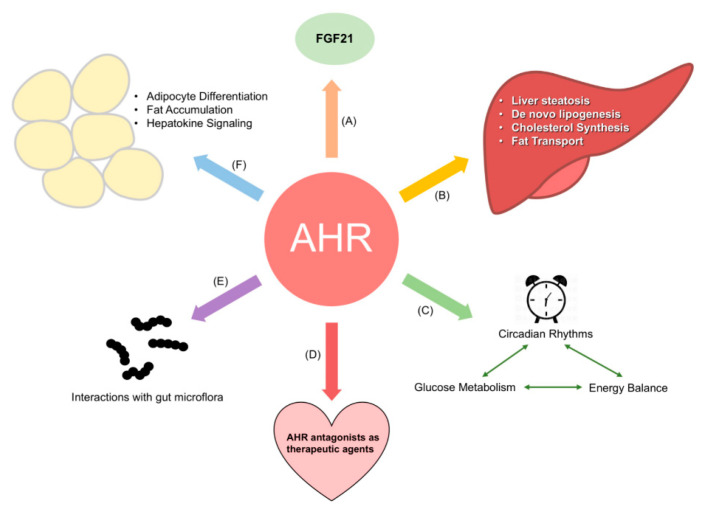
Current areas of research regarding the aryl hydrocarbon receptor (AHR) and energy metabolism. Current research interests include: (**A**) the role of the AHR in regulating FGF21 and adiposity; (**B**) how the AHR influences liver metabolism; (**C**) interactions between the AHR and circadian proteins, and their effect on glucose metabolism and energy balance; (**D**) the use of AHR antagonists to treat obesity and other metabolic disorders; (**E**) the relationship between the AHR, gut microflora, and the compounds excreted by these microorganisms; and (**F**) the role of the AHR in regulating adipocyte differentiation and adipogenesis.

While the Tomlinson laboratory worked on identifying the role of the AHR in obesity during the 2010s, the concurrent implication of the AHR in the physiological regulation of energy metabolism by the Xie and Tischkau laboratories in the early 2010s also led to a surge of interest into further teasing out the metabolic gene pathways that the AHR regulates in the absence of toxicants. Numerous laboratories are currently working to expand our understanding of the endogenous role of the AHR in energy balance (Reviewed in [91]). Areas currently under investigation are summarized in Figure 1, and include how AHR crosstalk with circadian genes affects metabolism [92], the role of the AHR in adipocytes [93], and interactions of the AHR with gut microflora and their effect on energy balance [57]. The remainder of this section shall discuss two of these in greater detail: the interaction of the AHR and fibroblast growth factor 21 (FGF21) and the relationship between the AHR, gut microbiota, and energy metabolism. 

In addition to Dr. Tischkau’s reports on the AHR-PPARα pathway in regulating energy balance, recent work has identified another key metabolic regulator that the AHR likely influences energy balance through, the fibroblast growth factor 21 (FGF21) hepatokine. FGF21 is a liver-secreted protein that enters circulation and binds to its cognate receptor in adipocytes to induce thermogenic gene expression [94,95]. FGF21 also appears to regulate sweet taste preference [96,97], influence energy metabolism through mechanisms involving the nervous system [98], and is the subject of intense study among pharmaceutical companies due to its ability to induce weight loss [99,100,101]. In 2014, Cheng et al. discovered that TCDD induced hepatic *Fgf21* expression in mice in an AHR-dependent manner [102]. This work was soon followed by another independent study in mice expressing CA-AHR [103], which likewise determined that the AHR can increase *Fgf21* transcription. Conversely, Girer et al. showed in 2016 that liver-specific deletion of the *Ahr* gene surprisingly increased *Fgf21* transcription in mice, and observed TCDD- and ICZ-driven transcriptional suppression of *Fgf21* in Hepa1c1c7 and primary human liver cells [104]. Girer and colleagues expanded upon these findings and determined that post-natal deletion of hepatic *Ahr* expression resulted in weight loss associated with increased brown fat and white fat respiration, but no increase in physical activity [105]. In this later study, TCDD was observed to transiently induce *Fgf21* gene expression, and resulted in distinct binding events at two different XRE sites within the *Fgf21* promoter region. While those data provide speculative evidence for XRE site-specific regulation of *Fgf21* gene expression, the ability for AHR agonists to both up-regulate and down-regulate *Fgf21* gene expression is not yet fully understood. Characterizing this phenomenon remains an important aspect of the involvement of the AHR in regulating metabolism given the clinical potential of the AHR-FGF21 regulatory axis not only in metabolism, but also in other diseases such as cancer [106,107].

Concurrent with the work conducted in Dr. Tomlinson’s and Dr. Tischkau’s laboratories to identify the role of the AHR in metabolism, other laboratories also began exploring how the gut microflora interact with intestinal AHR to influence host metabolism during the 2010s. As previously stated, the notion that bacteria in the gut could generate AHR ligands was already known by the 1990s. However, the difficulty of culturing these bacteria in the lab and the absence of advanced sequencing technologies precluded further advancements. Then in 2011, Marc Veldhoen and colleagues published their seminal findings that a loss of intestinal AHR activity reduced bacterial load and altered the composition of the microbiota [108]. Their work was quickly followed with a rapid expansion of research on this aspect of AHR biology. By 2014, researchers had uncovered several new endogenous AHR ligands produced by gut microbiota, demonstrated that tryptophan catabolites derived from gut microbiota influenced gut immunity homeostasis, and identified probiotic bacteria-derived compounds that could inhibit colitis [109,110,111]. Merely two years later, Korecka et al. importantly showed that these interactions of the gut microflora and the AHR are bi-directional, and that AHR-dependent modulations of the microbiome composition in the small intestine could impact host metabolic processes, such as hepatic fatty acid metabolism, and glucose metabolism in glucose-utilizing tissues [57]. As of writing, this branch of AHR biology continues to generate novel and insightful findings. For example, several laboratories are now identifying how the AHR, the gut microbiota, and the tryptophan metabolites they produce can be targeted to influence not only metabolic disease states [112,113,114,115], but neoplastic diseases as well [116,117]. For a more comprehensive review on the interaction of the AHR and the gut microflora, we refer the reader to another manuscript in this Special Issue of the International Journal of Molecular Sciences, entitled “How Ah Receptor Ligand Specificity Became Important in Understanding its Physiological Function” [118]. 

Revisiting our opening thoughts, each year brings more novel mechanisms of AHR activation to light. For example, Dr. Perdew’s group at the Pennsylvania State University group is actively exploring how AHR ligands can act independent of DNA binding [119,120], and have already provided evidence that the AHR can regulate fatty acid and cholesterol synthesis through XRE-independent activity [121,122] in the liver and the intestinal tract [123]. Concurrent work in Dr. Elferink’s laboratory at the University of Texas Medical Branch recently uncovered novel mechanisms of AHR action that work independent of its canonical DNA-binding partner, ARNT, and are working to differentiate ARNT-dependent and ARNT-independent aspects of ligand-activated AHR [3,124]. New discoveries such as these continually have us revisiting our understanding of how the AHR participates in physiological processes such as energy metabolism, and conceivably, additional novel mechanisms will be revealed in the coming years. In writing this review, we hope to have painted for the reader a clear picture of how toxicological and risk assessment studies that exposed adverse pathologies led to therapeutic endpoints, with an emphasis on metabolism and energy balance. The journey traced in this fascinating story highlights the dynamic nature of AHR research over the past 50 years, and underscores the potential for many more surprises.

## Data Availability

Data sharing not applicable.

## Abbreviations

2,4,5-T	2,4,5-trichlorophenol
ACACA	Acetyl-CoA carboxylase alpha
AHR	Aryl hydrocarbon receptor
αNF	Alpha-naphthoflavone
ARNT	Aryl hydrocarbon receptor nuclear translocator
CA	Constitutively-active
CYP1A1	Cytochrome P450 family 1 subfamily A member 1
FAS	Fatty acid synthase
FGF21	Fibroblast growth factor 21
I3C	Indole-3-carbinol
IDO1	Indoleamine-2,3-deoxygenase 1
KLF6	Kruppel-like Factor 6
Kyn	Kynurenine
PPAR	Peroxisome proliferator-activated receptor
SCD1	Stearoyl-CoA decarboxylase 1
SPP1	Secreted Phosphoprotein 1
TCDD	2,3,7,8-tetrachlorodibenzo-*p*-dioxin
